# IAT faking indices revisited: Aspects of replicability and differential validity

**DOI:** 10.3758/s13428-022-01845-0

**Published:** 2022-04-19

**Authors:** Jessica Röhner, Ronald R. Holden, Astrid Schütz

**Affiliations:** 1 Department of Psychology, University of Bamberg, Markusplatz 3, 96047 Bamberg, Germany; 2 Department of Psychology, Queen’s University, Kingston, ON Canada

**Keywords:** Implicit Association Test (IAT), Faking detection, Faking strategies, Faking indices, Machine learning

## Abstract

Research demonstrates that IATs are fakeable. Several indices [either slowing down or speeding up, and increasing errors or reducing errors in congruent and incongruent blocks; Combined Task Slowing (CTS); Ratio 150–10000] have been developed to detect faking. Findings on these are inconclusive, but previous studies have used small samples, suggesting they were statistically underpowered. Further, the stability of the results, the unique predictivity of the indices, the advantage of combining indices, and the dependency on how faking success is computed have yet to be examined. Therefore, we reanalyzed a large data set (*N* = 750) of fakers and non-fakers who completed an extraversion IAT. Results showed that faking strategies depend on the direction of faking. It was possible to detect faking of low scores due to slowing down on the congruent block, and somewhat less with CTS—both strategies led to faking success. In contrast, the strategy of increasing errors on the congruent block was observed but was not successful in altering the IAT effect in the desired direction. Fakers of high scores could be detected due to slowing down on the incongruent block, increasing errors on the incongruent block, and with CTS—all three strategies led to faking success. The results proved stable in subsamples and generally across different computations of faking success. Using regression analyses and machine learning, increasing errors had the strongest impact on the classification. Apparently, fakers use various goal-dependent strategies and not all are successful. To detect faking, we recommend combining indices depending on the context (and examining convergence).

Among implicit measures, one of the most prominent and valid is the Implicit Association Test (IAT; e.g., Bosson et al., 2000; Rudolph et al., 2008); however, numerous studies have demonstrated that the IAT (Greenwald et al., 1998) is fakeable (e.g., De Houwer et al., 2007; McDaniel et al., 2009; Röhner et al., 2011; Röhner & Lai, 2021; Steffens, 2004). Research on this issue has included both naïve faking (i.e., participants are not provided with information on faking strategies) and informed faking (i.e., participants are provided with information on faking strategies). Naïve faking has received a great deal of research attention and is a large concern for applied settings (e.g., Röhner & Holden, 2021) because this kind of faking does not require test respondents to have access to test-compromising information. Faking can result in altered test scores and rank orders—it can, thus, impact the validity of test scores (e.g., Salgado, 2016; see Röhner & Schütz, 2019, or Ziegler et al., 2012, for an overview). This has motivated researchers to develop faking indices that are able to identify fakers. Before we introduce these faking indices, we summarize the principle of the IAT.

## How does the IAT work?

The IAT is a computerized sorting task (Greenwald et al., 1998). It aims to assess the strength of implicit associations between two target concepts and an attribute dimension using participants’ reaction times during a categorization task. Participants sort stimuli into four different categories: two target categories and two attribute categories. For example, an extraversion IAT will include the target dimension self-relevant vs. other-relevant (e.g., me vs. other), and the attribute dimension of extraversion-related vs. introversion-related words (e.g., sociable vs. reticent). The IAT consists of seven blocks in total, of which Blocks 1, 2, and 5 are single or practice blocks that introduce the target or attribute discrimination. For these blocks, the categories of either the target concepts or the attribute concepts are presented in the upper corner of each side (left and right) of the computer screen. Participants respond to exemplars of each category by pressing a key. Blocks 3 and 4 and Blocks 6 and 7 are the so-called combined blocks, in which the attribute discrimination is paired with the target discrimination (i.e., participants must assign words from all four categories in these blocks). For Blocks 3 and 4 (i.e., the congruent blocks)^1^, participants are to respond to extraversion-related and self-relevant words with one key and to introversion-related and other-relevant words with the other key. For Blocks 6 and 7 (i.e., the incongruent blocks), participants are to respond to introversion-related and self-relevant words with one key and to extraversion-related and other-relevant words with the other key.^2^

The reasoning behind the IAT is that the sorting task should be simpler, and thus completed more quickly, if the two concepts that share one response key are strongly associated. If two concepts are weakly associated, sorting them into one category should be more difficult and should, therefore, be conducted more slowly. The IAT effect is computed as the difference in response times between the two combined blocks (in the example of the extraversion IAT: self linked with introversion minus self linked with extraversion) divided by their overall standard deviation. The IAT effect is used as an indicator of the strength of associations between the concepts (in the example of the extraversion IAT: self and extraversion as compared to self and introversion).

## Faking strategies that have been suggested to indicate faking on the IAT

The first studies to develop faking indices on the IAT analyzed empirical data and searched for evidence of *slowing down behavior*, something considered to be the most common faking strategy (e.g., Cvencek et al., 2010). Those studies only focused on slowing down behavior as a potential faking strategy (see Röhner et al., 2013) and suggested two indices that represented evidence of slowing down (see Table 1). Based on their research, Cvencek et al. (2010) recommended an index called Combined Task Slowing (i.e., CTS), whereas Agosta et al. (2011) advocated for an index called Ratio 150–10000.

**Table 1:** Faking strategies and faking indices by IAT block and faking goal

IAT block	Faking goal	
	Low scores	High scores
Faking indices that are based on conceptually derived faking strategies (Röhner et al., 2013)		
Congruent	*Slowing down on the congruent block* (i.e., Slow_Co; difference in reaction time between the congruent block under faking and the congruent block at baseline) *Increasing errors on the congruent block* (i.e., IncErr_Co; difference in errors between the congruent block under faking and the congruent block at baseline)	*Acceleration on the congruent block* (i.e., Accel_Co; difference in reaction time between the congruent block at baseline and the congruent block under faking) *Reducing errors on the congruent block* (i.e., RedErr_Co; difference in errors between the congruent block at baseline and the congruent block under faking)
Incongruent	*Acceleration on the incongruent block* (i.e., Accel_In; difference in reaction time between the incongruent block at baseline and the incongruent block under faking) *Reducing errors on the incongruent block* (i.e., RedErr_In; difference in errors between the incongruent block at baseline and the incongruent block under faking)	*Slowing down on the incongruent block* (i.e., Slow_In; difference in reaction time between the incongruent block under faking and the incongruent block at baseline) *Increasing errors on the incongruent block* (i.e., IncErr_In; difference between the incongruent block under faking and the incongruent block at baseline)
Faking indices that are based on slowing behavior		
Faster and slower	*CTS* (i.e., difference between the slower IAT block under faking and the faster IAT block under non-faking; Cvencek et al., 2010)	
Single and faster	*Ratio 150–10000* (i.e., ratio between the faster IAT block and the single IAT blocks under faking; Agosta et al., 2011)	

The content of this table is reprinted with permission from the publisher of Röhner et al. (2013) (10.1016/j.jrp.2013.02.009) under the CC-BY license (license number 5162330940652). Headers were amended according to this publication’s content.

### CTS

The CTS uses slowing down on the block in which a respondent was faster under non-faking conditions as an indicator of faking (Cvencek et al., 2010). Thus, the faster combined block of the baseline IAT is subtracted from the slower combined block of the faked IAT (i.e., *CTS* = *average reaction time of the slower combined block at faking* − *average reaction time of the faster combined block at baseline*). The procedure has been criticized because “[ …] given the plausible scenario that a test taker was faster on the congruent block in non-faking conditions and wants to fake higher scores, slowing down on the congruent block would lead to lower instead of higher scores and would thus be counterproductive” (see Röhner et al., 2013; p. 331). In the study of Röhner et al. (2013), the index accordingly identified naïve faking of low scores but did not identify naïve faking of high scores above chance levels (Röhner et al., 2013).^3^

### Ratio 150–10000

This procedure measures slowing down on the faster IAT block (i.e., congruent block) as compared to the single blocks (Agosta et al., 2011). It is calculated as follows: Only reaction times between 150 and 10,000 ms are used, and the others are excluded from further analyses. Errors are substituted with the mean of the corresponding IAT block with an added penalty of 600 ms. The average reaction time from the fastest combined block (i.e., either congruent or incongruent) is then divided by the average reaction time from the corresponding single blocks (i.e., Single Blocks 1 & 2, or Single Blocks 1 & 5; $\frac{average\ reaction\ time\ from\ the\ fastest\ combined\ block}{average\ reaction\ time\ from\ the\ corresponding\ single\ block}$). The index has been criticized because “[…] if a test taker wants to fake higher scores, slowing on that block [the congruent block] will not lead to the desired outcome, because the behavior produces lower scores” (see Röhner et al., 2013; p. 331). In the study of Röhner et al. (2013), that index detected neither faking of high scores nor faking of low scores above chance levels.

### Conceptually derived faking indices

Röhner et al. (2013) used a different procedure. Rather than analyzing empirical data and searching for empirical differences between fakers and non-fakers, Röhner et al. (2013) began by developing theory-based concepts of possible faking strategies and then operationalized and evaluated their performance. Thus, they took a deductive approach and analyzed which strategies could affect IAT effects. Furthermore, they considered faking strategies that extended beyond only *slowing down behavior*. They considered conceptually possible strategies that involved the manipulation of *reaction times* and *errors*.

Regarding the manipulation of reaction times, Röhner et al. (2013) noted that the IAT measures reaction times in categorizing sets of stimuli, and thus, IAT effects can be affected by specifically slowing down or speeding up on the combined blocks. Consequently, when the goal is to present categories as strongly associated and they share the same key, test takers may react more quickly in order to fake their scores in this direction, and when these categories require pressing different keys, they may react more slowly. Concerning errors, Röhner et al. (2013) noted that with the scoring algorithm to compute IAT effects that has been suggested by Greenwald et al. (2003a, 2003b), an error penalty is added to the reaction times of every trial in which an error is committed. Hence, the strategic reduction or enhancement of errors affects IAT effects.

In total, Röhner et al. (2013) derived four faking strategies for the faking of low scores and four faking strategies for the faking of high scores (see Table 1). In their research, they found that test respondents implemented different faking strategies depending on the direction of faking (i.e., faking strategies of faking low scores differed from those of faking high scores).

For the goal of faking *low scores*, Röhner et al. (2013) tested differences in reaction times between the congruent block during faking and the congruent block at baseline^4^ (i.e., slowing down behavior on the congruent block; Slow_Co), differences in reaction times between the incongruent block at baseline and the incongruent block during faking (i.e., acceleration behavior on the incongruent block; Accel_In), differences in errors between the congruent block during faking and the congruent block at baseline (i.e., a behavior of increasing errors on the congruent block; IncErr_Co), and differences in errors between the incongruent block at baseline and the incongruent block during faking (i.e., a behavior of avoiding errors on the incongruent block; RedErr_In).

For the faking of *high scores*, Röhner et al. (2013) investigated differences in reaction times between the incongruent block during faking and the incongruent block at baseline (i.e., slowing down behavior on the incongruent block; Slow_In), differences in reaction times between the congruent block at baseline and the congruent block during faking (i.e., acceleration behavior on the congruent block; Accel_Co), differences in errors between the incongruent block during faking and the incongruent block at baseline (i.e., a behavior of increasing errors on the incongruent block; IncErr_In), and differences in errors between the congruent block at baseline and the congruent block during faking (i.e., a behavior of avoiding errors on the congruent block; RedErr_Co).

## Status quo on faking indices

Agosta et al. (2011), Cvencek et al. (2010), and Röhner et al. (2013) have shown that faking on IATs can be detected via faking indices. Whereas Agosta et al. (2011) and Cvencek et al. (2010) focused only on slowing down behavior, Röhner et al. (2013) derived faking indices from conceptual faking strategies and provided insight into the complexity of faking detection on IATs.

### Faking strategies depend on faking direction

Röhner et al. (2013) demonstrated that slowing down is not the only faking strategy used by fakers. Fakers also tried to speed up and to manipulate errors in order to fake, which aligns with earlier findings of Steffens (2004) as well as Fiedler and Bluemke (2005). In addition, Röhner et al. (2013) demonstrated that the faking of high scores differs from the faking of low scores. Whereas fakers of low scores used slowing down on the congruent block, increasing errors on the congruent block, and Combined Task Slowing, fakers of high scores used acceleration on the congruent block. These results agree with recent research also showing that differences in faking behavior depend on the direction of faking (Bensch et al., 2019).

### Faking detection depends on faking direction

Faking strategies are reflected in faking indices, and Röhner et al. (2013) also demonstrated that faking detection depends on faking direction.^5^ Whereas slowing on the congruent block (Slow_Co), increasing errors on the congruent block (IncErr_Co), and Combined Task Slowing (CTS) were typical of naïve faking of low scores, acceleration on the congruent block (Accel_Co) was typical of naïve faking of high scores. These results are congruent with recent research showing that faking detection depends on the direction of faking (Röhner & Holden, 2021).

### Not all implemented faking strategies are related to faking success

Another important aspect is whether implemented faking strategies are indeed positively related to faking success (i.e., whether the IAT effect was changed as desired). Röhner et al. (2013) demonstrated that not all implemented faking strategies actually relate to faking success. For the implemented faking strategies, slowing on the congruent block (Slow_Co) was positively related to faking success under naïve faking of low scores, and acceleration on the congruent block (Accel_Co) was positively related to faking success under naïve faking of high scores. However, increased errors in the congruent block (IncErr_Co) and Combined Task Slowing (CTS) stood out because they were not significantly positively related to faking success for naïve faking of low scores. Thus, they represent unsuccessful faking attempts to naïvely fake low scores based on increased errors in the congruent block (IncErr_Co) or on Combined Task Slowing (CTS). Indeed, increasing errors on the congruent block was the faking strategy that worked best in Röhner et al. (2013; i.e.., it was able to classify 96% of the participants correctly as belonging to the faking or control group), indicating that this behavior is used by many fakers when attempting to fake (low). Nevertheless, this strategy was not related to faking success.

## Open questions: What we do not know yet

Although previous research has shed light on faking strategies and, thus, faking indices in IATs, there are several issues requiring further clarification. These issues should be investigated to determine which faking strategies are used by fakers and can be recommended for detecting faking on the IAT.

### Can previous findings be replicated using a high-powered sample?

A first, and probably the most prominent, issue in previous studies on faking indices is related to sample size and statistical power, because many of these studies have had low statistical power. This may explain why results have been inconclusive. Cvencek et al. (2010) had a minimum of *N* = 59 and a maximum of *N* = 82 participants, Agosta et al. (2011) had a minimum of *N* = 36 and a maximum of *N* = 72 participants, and Röhner et al. (2013) included *N* = 84 participants. Given that an acceptable level of discrimination in ROC curve analyses (receiver operating characteristic curves; Green & Swets, 1966) is an AUC (area under the curve) of .70 to .80 (see Hosmer & Lemeshow, 2000), and using a significance level of 0.05, post hoc power analyses using the R package pROC version 1.17.0.1 revealed power levels of > .75 (*N* = 59) to > .91 (*N* = 82) in the analyses of Cvencek et al. (2010), of > .52 (*N* = 36) to > .79 (*N* = 72) in the analyses of Agosta et al. (2011), and of > .77 in the analyses of Röhner et al. (2013). In addition to ROC curve analyses, previous studies have used multiple regression analyses (Cvencek et al., 2010), binary logistic regression analyses (Agosta et al., 2011), or correlation analyses and Fisher’s *z* test (Röhner et al., 2013). Cvencek et al. (2010) conducted a multiple regression analysis using five predictors and *N* = 47 to identify the best index (omnibus hypothesis). Using a significance level of 0.05, post hoc power analyses using G*Power 3.1.7 (Faul et al., 2009) reveal a power of .85 for detecting a large effect size, but a power of only .45 for detecting a medium effect size. Agosta et al. (2011) used binary logistic regression analyses on a total sample of 108. Data to compute power were requested from the original authors but were not available. Röhner et al. (2013) used correlation analyses with one predictor and 28 participants in each group (total *N* = 84). Using a significance level of 0.05, post hoc power analyses using G*Power 3.1.7 (Faul et al., 2009) revealed a power of .79 for detecting a large effect size, but a power of only .35 for detecting medium effect sizes. For Fisher’s *z* test, Röhner et al. (2013) selected a significance level of 0.10 to avoid being overly conservative and further, based on directional hypotheses, employed one-tailed testing. A post hoc power analysis using G*Power 3.1.7 (Faul et al., 2009) revealed a power of .80 for detecting large effect sizes, but a power of only .41 for detecting medium effect sizes.

Röhner et al. (2013) highlighted that their statistical tests may have been too conservative to detect medium effect sizes. Given several studies indicating that there is typically more evidence of faking when low scores are faked than when high scores are faked (e.g., Röhner et al., 2011; Viswesvaran & Ones, 1999), the results for faking high scores may be particularly less reliable. Further, with recent research demonstrating that medium effect sizes are most typical in psychological research, the problem of underpowered studies becomes especially relevant (Brysbaert, 2019). Given that the power of previous research is predominantly sufficient to detect only large effect sizes, these earlier investigations were generally statistically underpowered. As such, re-investigation of faking indices with larger samples that provide greater statistical power is warranted, and we decided to undertake replication analyses using a high-powered sample. In addition, there are several other issues that to date have not been sufficiently researched in previous studies on faking indices (see below) and, thus, we also included analyses to extend knowledge on these topics.

### Are the suggested faking strategies and, thus, faking indices stable across several samples?

A second issue is related to the stability of faking indices to detect faking across several samples—a property of faking indices which determines their usefulness. Cvencek et al. (2010) tested their faking index on several different IATs, and Agosta et al. (2011) tested their faking index on varying faking conditions (e.g., with vs. without training in IATs). However, because of the small sample sizes in this earlier research, it was not possible to investigate whether faking indices are reliable across several samples of the same IAT under the same conditions while achieving adequate statistical power. Thus, to date the fundamental issue of the stability of faking indices remains somewhat unanswered.

### What is the unique contribution of faking indices in detecting faking?

A third issue yet to be investigated concerns the joint effect of the recommended faking indices. What is the unique predictivity related to each faking index? Considering that all the suggested faking indices are based on reaction times and errors that are computed in different ways, an investigation of the unique contribution in prediction is particularly relevant. To date, this issue has not been addressed.

### What is the best way to combine the faking indices to achieve the best possible classification of fakers and non-fakers?

Considering that faking represents complex processes that can be accomplished by various pathways (see, e.g., Röhner et al., 2022), a fourth issue that to date has not been addressed is whether a combination of the suggested faking indices is superior for the detection of fakers relative to using a single faking index. If in the affirmative, what is the optimal combination?

### Are the results on faking success stable when effects of repeated measurement have been controlled for?

A last issue not addressed in previous studies concerns the assessment of faking success. In previous studies, faking success was computed either as the difference in the IAT effect between a baseline assessment and faking (i.e., *D* change; see Cvencek et al., 2010, and Röhner et al., 2013) or as a reversal in the IAT effect (i.e., from a positive IAT effect to a negative IAT effect; see Agosta et al., 2011). The attempt to use differences in the IAT effect between a baseline assessment and faking raises difficulties in interpretation because participants must take at least two IATs (i.e., baseline and faking), and repeated IAT administration has been shown to generate effects for repeated measurement (e.g., decreases in IAT effects; see for example Agosta et al., 2011; Connor & Evers, 2020; Schmitz, 2010). To control for repeated measurement effects, faking success could alternatively be computed in terms of an interaction effect (i.e., group × IAT effect of naïve faking/retest). Whether or not the results of previous studies could be confirmed when faking success is computed in this manner (i.e., when effects of repeated measurement are controlled for) has yet to be investigated.

## The present study

As one contribution, the present research is an attempt to replicate previous results with a high-powered sample. As a second contribution, our study goes beyond the issue of replicability by addressing open questions regarding the validity of faking indices. Therefore, we have both replication and extension portions included in this investigation.

### The replication portion

We followed the procedure of Röhner et al. (2013) to investigate whether the suggested indices (i.e., CTS, Ratio 150–10000, Slow_Co, Accel_Co, Slow_In, Accel_In, IncErr_Co, RedErr_Co, IncErr_In, and RedErr_In) can detect faking low scores and faking high scores in IATs. Because naïve faking represents the more common faking behavior in practical contexts, we focused on naïve faking in our analyses. We investigated faking on extraversion because of its usage in previous research and because both faking directions (i.e., faking high and faking low) are plausible with regard to this construct (e.g., McDaniel et al., 2009; Röhner et al., 2013; Röhner & Thoss, 2018; Steffens, 2004).

In contrast to previous studies, we used a large data set which thus had greater statistical power. Following Röhner et al. (2013), we first computed ROC curve analyses to investigate whether participants employed the faking strategies above chance. Second, also following Röhner et al. (2013), we ran correlation analyses between faking strategies and faking success and used Fisher’s *z* test to investigate whether faking strategies employed were more positively related to faking success than to effects of repeated measurement^6^ (because a strategy employed by fakers will not necessarily be successful in altering the IAT effect as desired). Given the greater statistical power in the current sample, we investigated the following hypotheses and evaluated whether we could replicate or extend the results of Röhner et al. (2013):

1. In line with previous research, we anticipated that faking detection in faking low and in faking high conditions would differ with respect to the faking indices. Based on previous results, the following outcomes were likely: Faking of low scores would be indicated by slowing down on the congruent block (Slow_Co), increased errors in the congruent block (IncErr_Co), and by Combined Task Slowing (CTS); faking of high scores would be indicated by acceleration on the congruent block (Accel_Co). However, given the small sample sizes and, relatedly, the barely sufficient statistical power of previous research, we surmised that findings from a highly powered test may provide somewhat different results. Because faking effects are smaller for faking high than for faking low (e.g., Röhner et al., 2011; Viswesvaran & Ones, 1999), these results may hold for faking low—but results on faking high may be different because, for faking low, the small sample sizes in previous studies had adequate power to detect the expected large faking effects whereas, for faking high, the small sample sizes did not have adequate power for detecting the expected moderate or even small effects.

Most likely fakers of high scores use strategies of slowing down, increasing errors, and Combined Task Slowing on the incongruent block, while fakers of low scores use them on the congruent block. Thus, alternatively, we expected the following: Whereas faking of low scores was anticipated to be indicated by slowing down on the congruent block (Slow_Co), increased errors in the congruent block (IncErr_Co), and by Combined Task Slowing (CTS), faking of high scores was expected to be indicated by slowing down on the incongruent block (Slow_In), increased errors in the incongruent block (IncErr_In), and by Combined Task Slowing (CTS).

(2) Based on previous research, we expected that not all strategies that are implemented by fakers are positively related to faking success. Given the small sample sizes and comparably low statistical power levels of previous research that was associated with correlation and regression analyses, it is plausible that results might change and that these changes again would be particularly associated with the faking of high scores (e.g., Röhner et al., 2011; Viswesvaran & Ones, 1999). Thus, we anticipated that the results for faking low would be replicated [i.e., for strategies that indicated faking low at levels above chance, slowing down on the congruent block (Slow_Co) and Combined Task Slowing (CTS) would be positively related to faking success, whereas increased errors in the congruent block (IncErr_Co) would not]. Further, slowing down on the congruent block (Slow_Co) and Combined Task Slowing (CTS) should be more positively related to faking success than to effects of repeated measurement. For faking high, we expected that slowing down on the incongruent block (Slow_In) and Combined Task Slowing (CTS) would be positively related to faking success, whereas increased errors in the incongruent block (IncErr_In) would not. Further, slowing down on the incongruent block (Slow_In) and Combined Task Slowing (CTS) should be more positively related to faking success than to effects of repeated measurement.

### The extension portion

To articulate further on the usefulness of faking indices, we investigated the stability of the results concerning the ability of faking indices to detect fakers. To do so, we randomly divided the overall data set into five subsamples^7^ and computed ROC curve analyses using the procedure described above (see Röhner et al., 2013).

(3) We expected that the faking indices would show stability with respect to their assignment of faking status. That is: faking indices that correctly classified whether participants belong to the faking group or to the control group in the overall data set would also correctly classify whether participants belong to the faking group or to the control group in the subsamples. In line with this reasoning, faking indices that *did not* correctly classify whether participants belonged to the faking group or to the control group in the overall data set would also *fail to* correctly classify whether participants belong to the faking group or to the control group in the subsamples.

We also extended previous research by exploring the unique contribution of the faking indices to the correct classification of fakers and non-fakers with multiple logistic regression analyses.

(4) Based on previous research that demonstrated increasing errors on the congruent block (in order to fake low scores) to be the strongest predictor in classifying participants as belonging to either the faking group or the non-faking group, and concerning the power issues (see above) that may impair the results concerning the faking of high scores, we expected that increasing errors on the congruent block (to fake low scores) and on the incongruent block (to fake high scores) would have the most impact on faking detection, including unsuccessful faking attempts (Röhner et al., 2013). We expected the other indices to have only small or even a negative impact on faking detection (Röhner et al., 2013).

Given that faking can be achieved by several pathways and combinations of behaviors, we extended previous research by using machine learning to identify the optimal combination of faking indices that increases the correct classification of fakers and non-fakers. Thus, we developed combinations of indices that were able to detect fakers above chance levels and explored their ability to correctly classify fakers and non-fakers using machine learning. In doing so, indices were differentially weighted and combined.

(5) We anticipated that there would be combinations of weighted indices that are superior to the use of single faking indices and explored which combinations work best.

In addition, we extended previous research findings and investigated whether the assessment of faking success as a difference in IAT effects between baseline assessment and faking, as done in earlier studies, might contaminate the effects of faking strategies on faking success because of repeated measurement effects in IATs (e.g., Agosta et al., 2011; Connor & Evers, 2020; Schmitz, 2010). Thus, we computed faking success in terms of an interaction effect (i.e., group × IAT effect of naïve faking/retest) and recalculated the correlation analyses and the Fisher’s *z* tests as described above.

(6) Because of repeated measurement effects in IATs that may lead to ambiguous interpretations concerning the relation of faking strategies and faking success when using difference scores to assess faking success, we expected that the effects of faking strategies on faking success would become more distinct when using a purer measure of faking success as an interaction effect.

## Method

### Data sets

To evaluate whether the abovementioned faking indices can detect fakers, we reanalyzed three unpublished data sets previously collected under the supervision of the first author in an investigation of faking on IATs measuring extraversion (data set 1, data set 2, and data set 3). For the analyses, we combined the data on the extraversion IATs from the three data sets. Thus, the final sample consisted of 750 participants (258 faking low, 245 control, 247 faking high; 576 women, 173 men, 1 no response; 744 students) with an average age of 22.05 years (*SD* = 4.07).

We chose the selected data sets for several reasons: First, data sets were from studies that included both faking high and faking low instructions. Because our interest was in the impact of faking high scores and of faking low scores, it was necessary that both faking directions were contained in the same data set. Second, with 750 participants included in these studies, power analyses using G*Power 3.1.7 (Faul et al., 2009) and R indicated high power for detecting medium effect sizes. Post hoc power analyses using G*Power 3.1.7 for detecting a medium effect size using an alpha level of .05 revealed a power > .99 for analyses of variance (ANOVAs) regarding the manipulation check analyses. Sensitivity analysis using G*Power 3.1.7 for a power of .95 and an alpha level of .05 revealed a minimum detectable effect size *f* of .07. Power analyses using the R package pROC version 1.17.0.1 revealed a power > .99 at a significance level of 0.05 for ROC curve analyses to detect an AUC associated with acceptable levels of discrimination (i.e., *AUC* = .70 to .80; see Hosmer & Lemeshow, 2000).^8^ Sensitivity analysis using the R package pROC version 1.17.0.1 revealed a minimum detectable AUC of .59.^9^ Power analyses using G*Power 3.1.7 for detecting a medium effect size using an alpha level of .05 revealed a power > .99 for correlation analyses.^10^ Sensitivity analysis using G*Power 3.1.7 for a power of .95 and an alpha level of .05 revealed a minimum detectable effect size *q* of .22.^11^ Power analyses using G*Power 3.1.7 for detecting a medium effect size using an alpha level of .05 revealed a power of > .95 for Fisher’s *z* test.^12^ Sensitivity analysis using G*Power 3.1.7 for a power of .95 and an alpha level of .05 revealed a minimum detectable effect size *q* of .30.^13^ Power analyses using the R package pwr version 1.3-0 revealed a power > .99 at a significance level of .001 for multiple logistic regression analyses to detect a medium effect size. Sensitivity analyses using the R package pwr version 1.3-0 for a power of .95 and an alpha level of .001 revealed a minimum detectable effect size *f*^*2*^ of .07. Third, in these data sets, participants worked on the extraversion IAT (i.e., assessing implicit extraversion) as well as on the extraversion scale (i.e., assessing explicit extraversion), allowing us to compute implicit–explicit correlations.

### Procedure

Participants took part in the studies in exchange for personal feedback and/or partial university course credit. In all studies, participants completed the extraversion IAT and the extraversion scale twice, with the IAT always preceding the self-report. On the first occasion (i.e., baseline), participants completed the IAT and the extraversion scale under standard instructions. On the second occasion, participants were randomly assigned to one of three conditions (i.e., control, faking high scores, or faking low scores). Participants in the control condition again responded under standard instructions on the IAT and on the extraversion scale. Fakers were asked to fake either high scores or low scores on the IAT and on the extraversion scale according to a personnel selection scenario. To assess the faking behavior of participants as would normally occur within an applied context, fakers were not provided with any strategies on how to fake (i.e., *naïve faking;* see Röhner et al., 2013). In the instructions for faking high scores, participants were asked to imagine they had been unemployed for one year and had now received a very attractive job offer. They were asked to fake high on extraversion to maximize the chances of being offered the job. The instructions for faking low scores included the description of a very unattractive job offer. However, because they received unemployment benefits, they had to apply and could not turn it down without risking loss of those benefits. To avoid being offered the job, participants were asked to fake low extraversion.

All measures, manipulations, and exclusions related to these data sets are reported and transparent. Measures that were included in the data collection but were not relevant for our hypotheses (and were therefore not part of our reanalyses) include the following: the Balanced Inventory of Desirable Responding (Musch et al., 2002), the Self-Monitoring Scale (Graf, 2004), and the Generalized Self-Efficacy Scale (Hinz et al., 2006) for data set 2; the Need for Cognition scale (Bless et al., 1994), the HEXACO-100 (Lee & Ashton, 2018), the Moral Attentiveness Scale (Pohling et al., 2014), the Moral Identity Scale (Aquino & Reed II, 2002), the Justice Sensitivity Inventory (Beierlein et al., 2012), and the need for cognition IAT (Fleischhauer et al., 2013) for data set 3. The manipulation was done as described in the procedure section. Participants were excluded based on Mahalanobis distances. The codes and results of these analyses are stored at the Open Science Framework (OSF) (https://osf.io/6vt7c/).

### Extraversion IAT

The extraversion IAT (Back et al., 2009) consisted of seven blocks of trials. The single-dimension practice Blocks, 1, 2, and 5, each included 24 trials. The combined Blocks, 3, 4, 6, and 7, each consisted of 48 trials. The IAT included the target discrimination between self-relevant (me, own, my, I, self) and other-relevant (you, others, they, your, them) words, and attribute discrimination between extraversion-related words (outgoing, talkative, active, sociable, impulsive) and introversion-related words (deliberate, reserved, shy, passive, reticent). Split-half reliability was .82 at baseline and .82 at naïve faking/retest (see Table 2 for split-half reliabilities at baseline and at naïve faking/retest on group levels).

**Table 2:** Descriptive statistics, and post hoc comparisons for the D scores of the extraversion IAT, split-half reliabilities, and implicit-explicit correlations

	Descriptive statistics and post hoc comparisons			Split-half reliabilities			Implicit-explicit correlations		
	Experimental group								
	Faking low	Control group	Faking high	Faking low	Control group	Faking high	Faking low	Control group	Faking high
Measurement occasion	*M* (*SD*)	*M* (*SD*)	*M* (*SD*)	*r*_*Split-Half*_	*r*_*Split-Half*_	*r*_*Split-Half*_	*r*_*ie*_	*r*_*ie*_	*r*_*ie*_
1 (Baseline)	−0.24 (0.44)_a1_	0.21 (0.41)_a1_	0.19 (0.39)_a1_	.85	.83	.78	.22**	.23**	.39**
2 (Naïve faking/retest)	−0.20 (0.53)_b2_	0.21 (0.42)_a1_	0.50 (0.48)_c2_	.66	.84	.82	.01	.21**	.17*

*N* = 750 (*n* faking low = 258, *n* control group = 245, and *n* faking high = 247); different alphabetic subscripts indicate significant differences between experimental groups (i.e., columns); different numeric subscripts identify significant differences between measurement occasions (i.e., rows) at *p* < .05, ** indicates *p* ≤ .001, * indicates *p* ≤ .050

Between participants, IATs were counterbalanced for combined block order to control for the finding that IAT effects tend to show stronger associations for the first paired categories (Schnabel et al., 2008). Within participants, the presentation of combined blocks was held constant (i.e., participants who worked on the congruent blocks before they worked on the incongruent blocks at baseline assessment had the same compatibility order with retest/faking and vice versa). Data from the combined blocks were used to compute IAT effects (*D*_1_ measure; Greenwald et al., 2003a, 2003b). Extremely long responses (i.e., more than 10,000 ms) were deleted. We computed the *D*_1_ measure with the R code provided by Röhner and Thoss (2019).

### Extraversion scale

Participants completed the respective scale from the NEO-Five Factor Inventory (Borkenau & Ostendorf, 2008; English version: Costa & McCrae, 1992). This scale consists of 12 items answered on five-point ratings that range from 1 (strongly disagree) to 5 (strongly agree). Scale characteristics and Cronbach’s alpha reliability, *M* = 28.35, *SD* = 6.70, and α = .82 at baseline, were comparable to Borkenau and Ostendorf’s (2008) values of *M* = 28.38, *SD* = 6.70, and α = .80.

### Analytic strategy

#### The replication portion

To replicate previous findings, we followed procedures articulated in Röhner et al. (2013) to evaluate whether CTS (Cvencek et al., 2010) and Ratio 150–10000 (Agosta et al., 2011), as well as Slow_Co, Accel_Co, Slow_In, Accel_In, IncErr_Co, RedErr_Co, IncErr_In, and RedErr_In (Röhner et al., 2013), were able to detect naïve faking of low and high scores. To compute the indices, we followed the procedures of Agosta et al. (2011), Cvencek et al. (2010), and Röhner et al. (2013). Thus, we used IAT data of the second measurement occasion (i.e., faking or retest) for the computation of Ratio 150–10000 (Agosta et al., 2011) and IAT data of the first (i.e., baseline) and second measurement occasion (i.e., faking or retest) for the computation of CTS (Cvencek et al., 2010), as well as Slow_Co, Accel_Co, Slow_In, Accel_In, IncErr_Co, RedErr_Co, IncErr_In, and RedErr_In (Röhner et al., 2013). Intercorrelations of the faking indices are shown in Tables 3 and 4.

**Table 3:** Intercorrelations of faking indices concerning faking low and control group

Faking indices	1	2	3	4	5	6
1. Slowing down on the congruent block	−	−.74**	.36**	−.29**	.98**	.07
2. Acceleration on the incongruent block	−.46**	−	−.34**	.42**	−.79**	.21**
3. Increasing errors on the congruent block	.17**	−.04	−	−.84**	.37**	−.01
4. Reducing errors on the incongruent block	−.14*	.11	−.32**	−	−.34**	−.03
5. CTS	.52**	−.64**	.11	−.15*	−	.08
6. Ratio 150–10000	.11	−.01	.09	−.11	−.04	−

The results for the faking low group (*n* = 258) are shown above the diagonal. The results for the control group (*n* = 245) are shown below the diagonal. *N* = 503. *p < .05. **p < .01

**Table 4:** Intercorrelations of faking indices concerning faking high and control groups

Faking indices	1	2	3	4	5	6
1. Slowing down on the incongruent block	−	−.25**	.52**	.13*	.86**	−.01
2. Acceleration on the congruent block	−.46**	−	.07	−.06	−.42**	−.23**
3. Increasing errors on the incongruent block	.11	−.14*	−	−.29**	.46**	−.18**
4. Reducing errors on the congruent block	−.04	.17**	−.32**	−	.06	.09
5. CTS	.64**	−.52**	.15*	−.11	−	−.04
6. Ratio 150–10000	.01	−.11	.11	−.09	−.04	−

The results for the faking high group (*n* = 247) are shown above the diagonal. The results for the control group (*n* = 245) are shown below the diagonal. *N* = 492. *p < .05. **p < .01

We conducted four kinds of analyses. First, we used an ANOVA with repeated measures on the extraversion IAT *D* scores as a manipulation check to investigate whether participants in the faking groups were able to fake the IAT.

Second, to examine which strategies were employed by fakers (and, therefore, which indices could detect faking) on the IAT, we used ROC curve analyses to evaluate how well each of the strategies was able to predict whether participants belonged to the control group or a faking group (see Röhner et al., 2013). If most fakers employed a specific strategy (e.g., slowing down on the congruent block to fake low scores), then it should be possible to differentiate fakers from non-fakers on the basis of that respective behavior. In ROC curve analyses, hit rates (for successfully identifying participants in the faking condition) are plotted as a function of false-alarm rates (falsely identifying respondents in the control group as fakers). The AUC indicates the success of each strategy in correctly predicting whether a participant belonged to the faking group or the control group (i.e., whether the faking status could be assigned at levels above chance). If the *AUC* was above and differed significantly from the .50 chance rate, the strategy was typically used by fakers, in contrast to non-fakers, at levels above chance. Analyses were performed separately for the faking high and faking low conditions (compared to the control group in each case).

Third, we conducted correlation analyses to evaluate the degree to which the strategies were connected to *faking success*.^14^ This is an important aspect to investigate because it is not necessarily the case that an employed strategy will also result in altering the IAT effects as desired. Faking success was computed as the difference in *D* scores between a faked and a non-faked IAT (*D* change; see e.g. Cvencek et al., 2010; Röhner et al., 2013) in order to allow comparisons of our results to the results of previous studies. Faking success for participants faking *low* scores was calculated by subtracting the *D* score of the faked IAT from the *D* score of the non-faked IAT completed at baseline assessment. Faking success for participants faking *high* scores was calculated by subtracting the *D* score of the non-faked IAT from the *D* score of the faked IAT. Thus, positive values indicate successful faking independent of the specific faking direction. We also computed *D* change for the control group (here, as the difference between the baseline assessment and repeated measurement to represent changes in *D* scores that were not due to the faking instructions; i.e., effects of repeated measurement).^15^

Fourth, we computed correlation analyses between *D* change and the respective faking strategy to assess the extent to which the use of a strategy was linked to success in changing the score. We used Fisher’s *z* test (Fisher, 1950) to compare the correlations in the faking groups to those in the control group to determine whether the correlations differed from effects of repeated measurement. We used *p <* .05 as significant in the Fisher’s *z* tests, and in line with Röhner et al. (2013), we employed one-tailed testing in the Fisher’s *z* tests.

#### The extension portion

In order to extend knowledge on the usefulness of IAT faking indices, we additionally computed the following four kinds of analyses. First, to investigate the stability of faking indices to detect fakers, we randomly assigned our sample into five subsamples and recalculated the ROC curve analyses as described above.

Second, extending previous research, we computed multiple logistic regression analyses to investigate the unique contribution of each faking index in the prediction of whether participants belonged in the control group or a faking group. Thus, we undertook two multiple logistic regressions, whereby we compared either participants in the faking low group with those in the control group, or participants in the faking high group with those in the control group. In each case, we used the respective faking indices (i.e., slowing down on the congruent block, acceleration on the incongruent block, increasing errors on the congruent block, reducing errors on the incongruent block, CTS, and Ratio 150–10000 when faking low was investigated, and slowing down on the incongruent block, acceleration on the congruent block, increasing errors on the incongruent block, reducing errors on the congruent block, CTS, and Ratio 150–10000 when faking high was investigated) as independent variables and predicted whether participants belonged to the faking group or control group. Odds ratios greater than 1 indicate that the unique contribution of the respective faking strategy of faking index was *positively* related to the detection of participants in the faking group. Odds ratios below 1 indicate that the unique contribution of the respective faking strategy of faking index was *negatively* related to the detection of participants in the faking group. We set *p <* .001 as the significance level in these analyses.

Third, extending previous research, we used machine learning to investigate combinations of faking indices in their ability to classify fakers and non-fakers correctly. We used an affine combination of the previously suggested faking indices that demonstrated their ability to detect fakers in our previous analyses (i.e., slowing down on the congruent block, increasing errors on the congruent block, and CTS when faking low scores, or slowing down on the incongruent block, increasing errors on the incongruent block, and CTS when faking high scores). Thus, we varied the individual weights of the indices from 0 to 1 with steps of 0.1 and summed the weighted indices to obtain new combined indices (i.e., 62 new combined indices for faking low and 62 new combined indices for faking high). We split the data into 80% training and 20% validation data sets and used the package groupdata2 to stratify those sets. We then trained decision trees using the package rpart and a tenfold cross-validation procedure (e.g., Orrù et al., 2020). We validated our results with the validation data sets. To assess whether the combined indices were superior to using single faking strategies and faking indices, we compared the resulting AUCs to those from the ROC curve analyses when computed with single indices.

Fourth, we recalculated the correlational analyses as described above but changed the method for assessing faking success in order to better control for repeated measurement effects. Thus, we computed faking success (for participants in the faking group) and effects of repeated measurement (for participants in the control group) as interaction effects, because repeated IAT administration may cause effects that confound the interpretation of findings (e.g., Agosta et al., 2011; Connor & Evers, 2020; Schmitz, 2010) when computed as a difference score (i.e., *D* change). Faking success for participants faking *low* scores as well as for participants faking *high* scores was computed as an interaction (i.e., group × *D* score of the faked IAT). We also computed the effect of repeated measurement for the control group in the same way. In each case, group was represented by effect coding (i.e., faking groups were coded as +1 and the control group was coded as −1). Note that due to this computation, successful faking thus far was *not independent* of the specific faking direction, because positive values indicated successful faking for fakers of high scores (i.e., participants had high scores at naïve faking) while negative values indicated successful faking for fakers of low scores (i.e., participants had low scores at naïve faking). Consequently, to facilitate interpretation, we multiplied the interaction effect by −1 for fakers of low scores and for the control group when compared against fakers of low scores so as to obtain positive values that indicate successful faking independent of the specific faking direction. We recalculated the Fisher’s *z* tests as described above but used the interaction effect to assess faking in order to control for effects of repeated measurement.

We stored all the data, the codes, and the results of these analyses at the OSF (https://osf.io/6vt7c/).

## Results

### Manipulation check

To examine whether the IATs could be faked, we conducted a 2 (measurement occasion: baseline vs. faking/retest) × 3 (experimental group) ANOVA with repeated measures on the extraversion IAT *D*_1_ scores. The main effects of measurement occasion, *F*(1, 747) = 5.45, *p =* 0.02, η^2^_partial_ = .01, ω^2^ = .01, and group, *F*(2, 747) = 50.51, *p <* .001, η^2^_partial_ = .12, ω^2^ = .12, were qualified by the expected significant and large interaction effect, *F*(2, 747) = 133.23, *p <* .001, η^2^_partial_ = .26, ω^2^ = .26.^16^ Thus, participants were able to fake high scores and low scores on the IAT (see Table 2). In agreement with previous research on faking (e.g., Salgado, 2016; see Röhner & Schütz, 2019, or Ziegler et al., 2012, for an overview), faking not only affected means, but was also related to changes in reliability and construct-related validity. Split-half reliabilities and correlations between the extraversion IAT and the extraversion scale (i.e., implicit–explicit correlations) are presented in Table 2.

### What fakers do and what leads to faking success: Faking low

#### Expected strategies

According to the conceptual approach of Röhner et al. (2013), possible strategies to fake low scores are to slow down on the congruent block, react faster on the incongruent block, increase the number of errors on the congruent block, or reduce the number of errors on the incongruent block. Based on Cvencek et al. (2010), faking low should be indicated by slower reaction times on the combined block on which participants had reacted more quickly under non-faking (i.e., as represented by the CTS index). According to Agosta et al. (2011), faking low would stand out by the participants’ slower reaction times on the congruent block compared to single blocks (i.e., as represented by the index Ratio 150–10000).

#### What fakers actually did

**Fig. 1:**
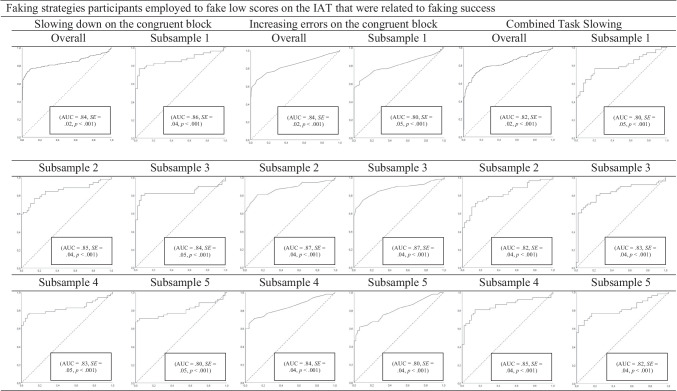

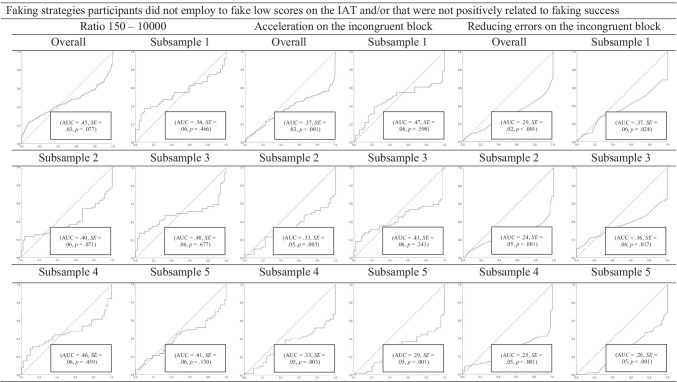
The upper part of the figure shows the ROC curve analyses (Green & Swets, 1966) for the faking strategies participants employed to fake low scores on the IAT that were related to faking success. The lower part of the figure shows the ROC curve analyses for the faking strategies participants did not employ to fake low scores on the IAT and/or that were not positively related to faking success. Overall = results of the ROC curve analyses concerning the overall sample. Subsamples 1 to 5 = results of the ROC curve analyses concerning the respective subsamples (i.e., 1, 2, 3, 4, and 5). The hit rate (proportion of correctly identified faking participants) is plotted on the *y*-axis against the false alarm rate (proportion of non-faking participants incorrectly identified as fakers) on the *x*-axis. The diagonal line represents chance success. The area under the curve (AUC) corresponds to the percentage correct on a two-alternative forced-choice detection task.

ROC curve analyses (Table 5; Fig. 1) indicated that naïve participants who faked low scores could be distinguished from non-fakers via slowing down on the congruent block, increasing errors on the congruent block, and Combined Task Slowing (*AUCs* = .84, .84, and 82, respectively). ROC curve analyses also revealed that acceleration on the incongruent block and reducing errors on the incongruent block were a typical sign of non-fakers, not fakers, because *AUC*s were significantly below the chance rate of .50 (*AUCs* = .37 and .29, respectively). This result could be explained by practice effects of non-fakers (i.e., they were able to speed up and to commit fewer errors due to practice) that are especially pronounced on incongruent blocks (see Fiedler & Bluemke, 2005). The Ratio 150–10000 index did not detect faking above chance levels (i.e., above 50%; *AUC* = .45).

**Table 5:** Implementation and success of faking strategies and indices concerning faking low

Faking strategies and indices	Implementation		Correlation with											
			Faking success			Effects of repeated measurement			Faking success			Effects of repeated measurement		
			When computed as *D* change						When computed as Interaction Effect					
	*AUC*	*SE*	*r*	*p*	*n*	*r*	*p*	*n*	*r*	*p*	*n*	*r*	*p*	*n*
Slowing down on the congruent block	**.84**	.02	**.51**	< .001	253	.36	< .001	241	**.50**	< .001	253	-.03	.686	241
Acceleration on the incongruent block	.37	.03	.41	< .001	256	.47	< .001	243	**.33**	< .001	256	-.07	.308	253
Increasing errors on the congruent block	**.84**	.02	.03	.587	258	.20	.001	243	.04	.580	258	-.13	.050	243
Reducing errors on the incongruent block	.29	.02	**.23**	< .001	258	.05	.422	244	**.24**	< .001	258	-.09	.173	244
CTS	**.82**	.02	**.17**	.006	253	-.11	.089	243	.21	.001	253	.25	< .001	243
Ratio 150–10000	.45	.03	-.12	.055	257	.15	.022	245	-.10	.111	257	-.21	.001	245

*AUC*s in bold indicate that the strategy- or index-classified participants as belonging to the control or faking low group at levels above chance (> .50). Faking success = changes in IAT effects according to faking instructions. Effects of repeated measurement = changes in IAT effects in the control group (i.e., not due to faking instructions). Correlations printed in bold indicate that the significant positive correlation between the relevant faking strategy or faking index and faking success in the faking low group was significantly higher than the correlation between the respective behavior and effects of repeated measurement in the control group according to Fisher’s *z* tests at *p* < .05.

##### Stability of findings

Examination of the stability of prediction in the ROC curve analyses on subsamples clearly demonstrates that the faking indices that were able to detect faking low above chance levels in the overall sample were also able to detect faking low above chance levels in the subsamples. Further, the faking indices that were not able to detect faking low above chance levels in the overall sample were also not able to detect faking low above chance levels in the subsamples (see Fig. 1).^17^

##### Unique contributions of faking strategies

The results of the multiple logistic regression model demonstrate that faking strategies allow for the correct classification of fakers (of low scores) and non-fakers (see Table 6). However, the unique contribution of faking strategies in predicting faking differed. The strongest predictor of faking was increasing errors on the congruent block. If this strategy increased by 1, the probability of belonging to the class of fakers increased by about 25%. Slowing down on the congruent block had a small impact. If this strategy increased by 1, the probability of belonging to the class of fakers increased by about 1%. CTS and acceleration on the incongruent block had no significant contribution to the prediction. That is, the odds of belonging to fakers or non-fakers were equal. Reducing errors on the incongruent block had a small negative contribution to the prediction of faking. If this strategy increased by 1, the probability of belonging to the class of fakers decreased by about 17%. Ratio 150–10000 had a strong negative impact on the prediction of faking. If Ratio 150–10000 increased by 1, the probability of belonging to the class of fakers decreased by about 68%. However, the confidence interval implies that the relationship could be either positive or negative.

**Table 6:** Logistic regression for implementation of faking strategies and indices concerning faking low

Faking strategies and indices	*B*	95 % CI for odds ratio			*SE* (*B*)	*R*^2^		
		*LL*	*Odds ratio*	*UL*		*H-L*	*C-S*	*Na*
						.47	.48	.64
Constant	−0.05	0.11	0.95	8.50	1.11			
Slowing down on the congruent block	0.01***	1.00	1.01	1.01	0.00			
Increasing errors on the congruent block	0.22***	1.14	1.25	1.39	0.05			
CTS	0.00	1.00	1.00	1.00	0.00			
Ratio 150–10000	−1.14	0.04	0.32	2.37	1.03			
Acceleration on the incongruent block	0.00	1.00	1.00	1.00	0.00			
Reducing errors on the incongruent block	−0.18***	0.75	0.83	0.92	0.05			

CI = confidence interval; *LL* = lower limit; *UL* = upper limit; *H-L* = Hosmer-Lemeshow; *C-S* = Cox-Snell; *Na* = Nagelkerke; Model 𝜒^2^(6) = 329.94, *p* < .001. ***p < .001

##### Combined faking strategies

**Fig. 2:**
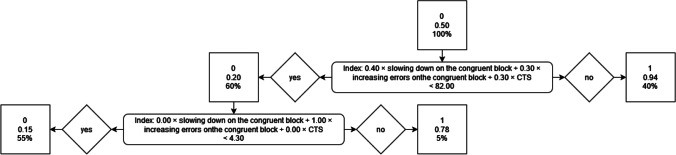
The final decision tree with combined indices regarding the faking of low scores

The results of the final decision tree can be seen in Fig. 2. The two relevant combined faking strategies are (1) 0.40 × slowing down on the congruent block + 0.30 × increasing errors on the congruent block + 0.30 × CTS, and (2) 0.00 × slowing down on the congruent block + 1.00 × increasing errors on the congruent block + 0.00 × CTS. The decision tree is composed of two steps. In a first step, participants with values above 82.00 in 0.40 × slowing down on the congruent block + 0.30 × increasing errors on the congruent block + 0.30 × CTS are classified as being fakers, whereas those with values below 82.00 in 0.40 × slowing down on the congruent block + 0.30 × increasing errors on the congruent block + 0.30 × CTS are further evaluated in a second step. In a second step, the remaining participants that have values above 4.30 in 0.00 × slowing down on the congruent block + 1.00 × increasing errors on the congruent block + 0.00 × CTS are classified as being fakers, whereas those with values below 4.30 in 0.00 × slowing down on the congruent block + 1.00 × increasing errors on the congruent block + 0.00 × CTS are classified as being non-fakers. The corresponding *AUC* is 0.85 (*SE* = 0.04), *p* ≤ .001, and thus is somewhat higher than the results of the ROC curve analyses concerning the overall sample.

#### Which strategies were successful?

##### Faking success as *D* change

Correlation analysis (see Table 5) between slowing down on the congruent block and faking success revealed that this strategy was strongly and positively related to faking success when faking success was computed as *D* change (see Cvencek et al., 2010; Röhner et al., 2013). Additionally, Fisher’s *z* tests revealed that slowing down on the congruent block was more strongly correlated with faking success in the faking (low) group than with effects of repeated measurement in the control group when using *D* change (see Cvencek et al., 2010; Röhner et al., 2013), Fisher’s *z* = 2.05, *p =* .002. Although it was used by fakers of low scores, the strategy to increase errors on the congruent block was not significantly related to faking success, but was to a small (positive) extent related to effects of repeated measurement in the control group when using *D* change, Fisher’s *z* = −1.92, *p =* .027. Correlation analyses showed that CTS was to a small degree positively related to faking success when faking success was computed as *D* change (Table 5). Fisher’s *z* test showed that CTS was more strongly correlated with faking success in the faking (low) group than with effects of repeated measurement in the control group using *D* change, Fisher’s *z* = 3.12, *p =* .001.

Correlation analyses also demonstrated a moderate association of acceleration on the incongruent block and a small association of reducing errors on the incongruent block with faking success when faking success was computed as *D* change. Fisher’s *z* tests, however, revealed that only error reduction on the incongruent block was more strongly correlated with faking success in the faking (low) group than with effects of repeated measurement in the control group when faking success was computed as *D* change, Fisher’s *z* = 2.05, *p =* .002. Acceleration on the incongruent block was correlated at comparable levels with faking success in the faking (low) group and with effects of repeated measurement in the control group when using *D* change, Fisher’s *z* = 0.83, *p =* .204.

Correlation analyses indicated that the behavior measured by Ratio 150–10000 was negatively (at a descriptive level) related to faking success when faking success was computed as *D* change and, thus, is even counterproductive. Moreover, Ratio 150–10000 was more strongly correlated with effects of repeated measurement in the *D* score in the control group than with faking success in the faking (low) group when computed as *D* change, Fisher’s *z* = −3.03, *p =* .001.

##### Faking success as an interaction effect

Correlation analysis (see Table 5) between slowing down on the congruent block and faking success revealed that this strategy was strongly and positively related to faking success when faking success was computed as an interaction effect. Additionally, Fisher’s *z* tests revealed that slowing down on the congruent block was more strongly correlated with faking success in the faking (low) group than with effects of repeated measurement in the control group when analyzing the interaction effect, Fisher’s *z* = 6.40, *p* ≤ .001. Although used by fakers of low scores, the strategy to increase errors on the congruent block was not significantly related to faking success but was to a small and negative extent related to effects of repeated measurement in the control group when analyzing an interaction effect, Fisher’s *z* = 1.92, *p =* .029. Correlation analyses showed that CTS was to a small degree positively related to faking success when faking success was analyzed as an interaction effect (Table 5). Fisher’s *z* test revealed that CTS correlated with effects of repeated measurement in the control group at a comparable level as faking success in the faking (low) group when analyzing an interaction effect, Fisher’s *z* = −0.47, *p =* .320.

When computing faking success as an interaction effect, correlational analyses demonstrated a moderate positive association of acceleration on the incongruent block and a small positive association of reducing errors on the incongruent block with faking success. Fisher’s *z* tests revealed that acceleration on the congruent block was more strongly correlated with faking success in the faking (low) group than with effects of repeated measurement in the control group when analyzing an interaction effect, Fisher’s *z* = 4.63, *p* ≤ .001, and error reduction on the incongruent block was more strongly correlated with faking success in the faking (low) group than with effects of repeated measurement in the control group when analyzing an interaction effect, Fisher’s *z* = 3.73, *p* ≤ .001.

Correlation analyses indicated that the behavior measured by Ratio 150–10000 was negatively (at a descriptive level) related to faking success when faking success was analyzed as an interaction effect and, thus, is even counterproductive. Moreover, Ratio 150–10000 was correlated at comparable levels with faking success in the faking (low) group and with effects of repeated measurement in the control group when analyzing the interaction effect, Fisher’s *z* = 1.26, *p =* .105.

### What fakers do and what leads to faking success: Faking high

#### Expected strategies

According to the conceptual approach of Röhner et al. (2013), possible strategies to fake high scores are to slow down on the incongruent block, react faster on the congruent block, increase the number of errors on the incongruent block, or reduce the number of errors on the congruent block. According to Agosta et al. (2011) and Cvencek et al. (2010), faking high would be indicated by the same faking behavior as faking low.

#### What fakers actually did

**Fig. 3:**
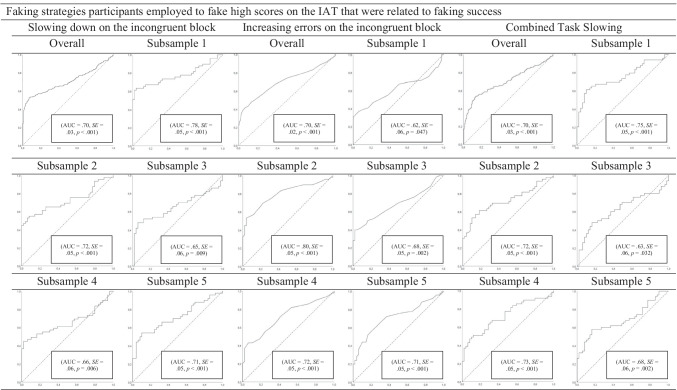

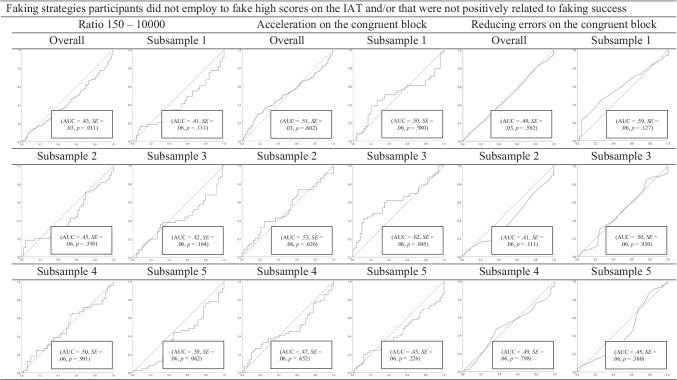
The upper part of the figure shows the ROC curve analyses (Green & Swets, 1966) for the faking strategies participants employed to fake high scores on the IAT that were related to faking success. The lower part of the figure shows the ROC curve analyses for the faking strategies that participants did not employ to fake high scores on the IAT and/or that were not positively related to faking success. Overall = results of the ROC curve analyses concerning the overall sample. Subsamples 1 to 5 = results of the ROC curve analyses concerning the respective subsamples (i.e., 1, 2, 3, 4, and 5). The hit rate (proportion of correctly identified faking participants) is plotted on the *y*-axis against the false alarm rate (proportion of non-faking participants incorrectly identified as fakers) on the *x*-axis. The diagonal line represents chance success. The area under the curve (AUC) corresponds to the percentage correct on a two-alternative forced-choice detection task

ROC curve analyses (Table 7; Fig. 3) revealed that participants could be classified correctly as belonging to the faking (high) group or control group via slowing down on the incongruent block, increasing errors on the incongruent block (*AUCs* = .70 and .70, respectively), and Combined Task Slowing (*AUC =* .70). Participants could not be classified correctly as belonging to the faking (high) group or control group via the strategies to accelerate on the congruent block (*AUC* = .51) or to reduce errors on the congruent block (*AUC* = .49; see also Table 7 and Fig. 3). As was true with faking low, the Ratio 150–10000 index did not correctly classify participants in the faking (high) and control groups. The *AUC* of .43 was significantly below the chance level, indicating that the behavior here was more typical for non-fakers.

**Table 7:** Implementation and success of faking strategies and indices concerning faking high

Faking strategies and indices	Implementation		Correlation with											
			Faking success			Effects of repeated measurement			Faking success			Effects of repeated measurement		
			When computed as *D* change						When computed as Interaction Effect					
	*AUC*	*SE*	*r*	*p*	*n*	*r*	*p*	*n*	*r*	*p*	*n*	*r*	*p*	*n*
Slowing down on the incongruent block	**.70**	.02	**.64**	< .001	246	.47	< .001	243	**.53**	< .001	246	−.07	.308	243
Acceleration on the congruent block	.51	.03	.41	< .001	244	.36	< .001	241	**.15**	.019	244	−.03	.686	241
Increasing errors on the incongruent block	**.70**	.02	**.37**	< .001	246	.05	.422	244	**.37**	< .001	246	−.09	.171	244
Reducing errors on the congruent block	.49	.03	.16	.016	236	.20	.001	243	**.19**	.004	236	−.13	.050	243
CTS	**.70**	.02	**.36**	< .001	246	.11	.089	243	**.50**	< .001	246	−.25	< .001	243
Ratio 150–10000	.43	.03	−.13	.050	245	−.15	.022	245	−.25	< .001	245	.21	.001	245

*AUC*s in bold indicate that the strategy or index classified participants as belonging to the control or faking high group at levels above chance (> .50). Faking success = changes in IAT effects according to faking instructions. Effects of repeated measurement = changes in IAT effects in the control group (i.e., not due to faking instructions). Correlations printed in bold indicate that the significant positive correlation between the relevant faking strategy or faking index and faking success in the faking high group was significantly higher than the correlation between the respective behavior and effects of repeated measurement in the control group according to Fisher’s *z* tests at *p* < .05.

##### Stability of findings

Consideration of the stability of prediction in the ROC curve analyses on subsamples clearly demonstrates that the faking indices that were able to detect faking high above chance levels in the overall sample were also able to detect faking high above chance levels in the subsamples, and that the faking indices that were not able to detect faking high above chance levels in the overall sample were also not able to detect faking high above chance levels in the subsamples (see Fig. 3).^18^

##### Unique contributions of faking strategies

The results of the multiple logistic regression model demonstrate that faking strategies allow for the correct classification of fakers (of high scores) and non-fakers (see Table 8). However, the unique contribution of faking strategies in predicting faking differed. The strongest predictor of faking was increasing errors on the incongruent block. If this strategy increased by 1, the probability of belonging to the class of fakers increased by about 17%. Slowing down on the incongruent block, CTS, and acceleration on the congruent block had no significant contribution to the prediction. That is, the odds of belonging to fakers or non-fakers were equal. Reducing errors on the congruent block had a small negative contribution to the prediction of faking. If this strategy increased by 1, the probability of belonging to the class of fakers decreased by about 6%. Ratio 150–10000 had a strong negative impact on the prediction of faking. If Ratio 150–10000 increased by 1, the probability of belonging to the class of fakers decreased by about 67%. However, the confidence intervals concerning both strategies imply that the relationship can be either positive or negative.

**Table 8:** Logistic regression for implementation of faking strategies and indices concerning faking high

Faking strategies and indices	*B*	95 % CI for odds ratio			*SE* (*B*)	*R*^2^		
		*LL*	*Odds ratio*	*UL*		*H-L*	*C-S*	*Na*
						.20	.24	.32
Constant	0.39	0.18	1.48	12.50	1.08			
Slowing down on the incongruent block	0.00	1.00	1.00	1.00	0.00			
Increasing errors on the incongruent block	0.16***	1.09	1.17	1.27	0.04			
CTS	0.00	1.00	1.00	1.00	0.00			
Ratio 150–10000	−1.10	0.05	0.33	2.40	1.01			
Acceleration on the congruent block	0.00	1.00	1.00	1.00	0.00			
Reducing errors on the congruent block	−0.06	0.86	0.94	1.02	0.04			

CI = confidence interval; *LL* = lower limit; *UL* = upper limit; *H-L* = Hosmer-Lemeshow; *C-S* = Cox-Snell; *Na* = Nagelkerke; Model 𝜒^2^(6) = 136.89, *p* < .001. ***p < .001

##### Combined faking strategies

**Fig. 4:**
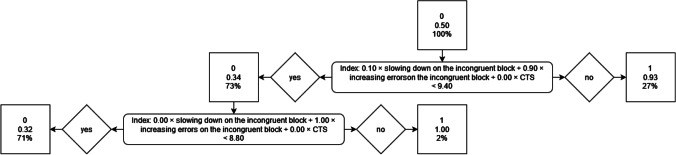
The final decision tree with combined indices regarding the faking of high scores

The results of the final decision tree can be seen in Fig. 4. The two relevant combined faking strategies are (1) 0.10 × slowing down on the incongruent block + 0.90 × increasing errors on the incongruent block + 0.00 × CTS, and (2) 0.00 × slowing down on the incongruent block + 1.00 × increasing errors on the incongruent block + 0.00 × CTS. The decision tree is composed of two steps. In a first step, participants with values above 9.40 in 0.10 × slowing down on the incongruent block + 0.90 × increasing errors on the incongruent block + 0.00 × CTS are classified as being fakers, whereas those with values below 9.40 in 0.10 × slowing down on the incongruent block + 0.90 × increasing errors on the incongruent block + 0.00 × CTS are further evaluated in a second step. In a second step, the remaining participants that have values above 8.80 in 0.00 × slowing down on the congruent block + 1.00 × increasing errors on the congruent block + 0.00 × CTS are classified as being fakers, whereas those with values below 8.80 in 0.00 × slowing down on the congruent block + 1.00 × increasing errors on the congruent block + 0.00 × CTS are classified as being non-fakers. The corresponding *AUC* is 0.71 (*SE* = 0.04), *p* ≤ .001, and thus is somewhat higher than the results of the ROC curve analyses concerning the overall sample.

#### Which strategies were successful?

##### Faking success as *D* change

Correlation analyses indicated that slowing down on the incongruent block was strongly and significantly positively correlated with faking success, and that increasing errors on the incongruent block and Combined Task Slowing were each moderately and significantly positively correlated with faking success (Table 7). Moreover, all three of these strategies were more strongly correlated with faking success in the faking (high) group than with effects of repeated measurement in the control group (Fisher’s *z* = 2.73, *p =* .003, for slowing down on the incongruent block; Fisher’s *z* = 3.72, *p* ≤ .001, for increasing errors on the incongruent block; Fisher’s *z* = 2.93, *p =* .002, for CTS).

Acceleration on the congruent block was moderately related to faking success, while reducing errors on the congruent block was only to a small extent associated with faking success. Both behaviors, however, were also about comparably related to *D* change in the control group, which could be due to practice effects (cf. Fiedler & Bluemke, 2005), Fisher’s *z* = 0.64 (*p =* .260) for acceleration on the congruent block, and Fisher’s *z* = −0.45 (*p =* .327) for reducing errors on the congruent block. The index of Agosta et al. (2011) was about equally and even negatively correlated with faking success in the faking (high) group in comparison with the effects of repeated measurement in the control group (Fisher’s *z* = 0.22, *p =* .411).

##### Faking success as an interaction effect

Correlation analyses indicated that slowing down on the incongruent block and Combined Task Slowing were each strongly and significantly positively correlated with faking success, and that increasing errors on the incongruent block was moderately and significantly positively correlated with faking success when faking success was analyzed as an interaction effect (Table 7). Moreover, all three of these strategies were more strongly correlated with faking success in the faking (high) group than with effects of repeated measurement in the control group (Fisher’s *z* = 7.26, *p* ≤ .001, for slowing down on the incongruent block; Fisher’s *z* = 8.84, *p* ≤ .001, for CTS; Fisher’s *z* = 5.27, *p* ≤ .001, for increasing errors on the incongruent block).

Acceleration on the congruent block and reducing errors on the congruent block were each to a small extent related to faking success. Both behaviors were more strongly correlated with faking success in the faking (high) group than with effects of repeated measurement in the control group (Fisher’s *z* = 1.98, *p =* .024, for acceleration on the congruent block, and Fisher’s *z* = 3.51, *p* ≤ .001, for reducing errors on the congruent block). The index of Agosta et al. (2011) was negatively correlated with faking success in the faking (high) group and positively correlated with effects of repeated measurement in the control group (Fisher’s *z* = −5.62, *p* ≤ .001).

## Discussion

Because of concerns about low statistical power in previous studies, we reanalyzed a large data set to conduct high-powered analyses of previously suggested IAT faking indices: CTS (Cvencek et al., 2010), Ratio 150–10000 (Agosta et al., 2011), Slow_Co, Accel_Co, Slow_In, Accel_In, IncErr_Co, RedErr_Co, IncErr_In, and RedErr_In (Röhner et al., 2013). With this replication, our research aimed to extend previous findings by shedding light on whether these results are stable across subsamples, on the unique contribution of faking indices in faking detection, on the advantage of combined faking indices, and on the independence of results with regard to how faking success is computed.

Results indicated that fakers of low scores and fakers of high scores use different faking strategies. Faking of low scores could be detected via slowing down on the congruent block, increasing errors on the congruent block, and somewhat less with Combined Task Slowing, whereas faking of high scores could be detected at comparable levels via slowing down on the incongruent block, increasing errors on the incongruent block, and with Combined Task Slowing. These results showed stability in all subsample analyses, pointing to their robustness. In general, increasing errors had the most impact for detecting participants aiming to fake low scores and high scores. The (relative) importance of increasing errors as a strategy was also underscored by machine learning, indicating that combining several faking indices somewhat improved prediction. Nevertheless, increasing errors was associated with the highest weight for prediction.

Not all strategies that indicated faking at levels above chance led to faking success. The pattern of results also depends on how faking success is computed. Following past research to use *D* change to quantify faking success, successful fakers of low scores were detected because they slowed down on the congruent block and showed Combined Task Slowing. Although fakers of low scores used the strategy of increasing errors on the congruent block, this strategy did not lead to faking success. In computing faking success as an interaction, the results in general point to a similar interpretation, but only concerning the theoretically deduced faking strategies of slowing down and increasing errors on the congruent block. Combined Task Slowing was not indicative of successful faking (beyond effects of repeated measurement) when faking success was computed as an interaction effect. Successful fakers of high scores were detected because they slowed down on the incongruent block, increased errors on the incongruent block, and showed Combined Task Slowing. This pattern emerged irrespective of how faking success was computed. Thus, to detect faking on the IAT, it is important to include multiple faking indices and to evaluate their convergence. In addition, the definition of faking success should be considered in this evaluation.

### Fakers use different faking strategies when faking low scores than when faking high scores

Our results highlight that fakers use different faking strategies with respect to the direction of faking. Whereas fakers of low scores slowed down and increased errors on the congruent block, fakers of high scores slowed down and increased errors on the incongruent block. Thus, although slowing down and increasing errors were used by both groups, groups differed with respect to the IAT block they manipulated (fakers of low scores focused on the congruent block, whereas fakers of high scores focused on the incongruent block). This may also explain why the Combined Task Slowing behavior (CTS), that does not control for which blocks are computed with each other, performs better regarding faking low than faking high. Another intriguing point is that acceleration on the incongruent block and reducing errors on the incongruent block, which have been theoretically considered to be employed for faking low, were typical signs of non-fakers. Furthermore, acceleration on the congruent block and reducing errors on the congruent block, which have been theoretically considered to be employed for faking high scores, were not used by fakers at levels above chance. Thus, not all strategies that are possible are actually used in order to fake. This result highlights the importance of empirically testing faking strategies.

### Not all faking indices work at levels above chance

In principle, acceleration on the incongruent block and reducing errors on the incongruent block could be used to identify non-fakers, and thus indirectly also identify fakers of low scores (because sorting out non-fakers will also identify fakers). In contrast, acceleration on the congruent block and reducing errors on the congruent block could detect fakers of high scores only at chance levels. The Ratio 150–10000 index was unable to identify fakers above chance levels in faking low conditions and indicated non-fakers when faking high was the goal. Agosta et al. (2011) suggested their index on the basis of analyses with the autobiographical IAT (aIAT). Because the aIAT measures autobiographical content whereas *traditional* IATs measure non-biographical content but aspects of personality, attitudes, and stereotypes, the index of Agosta et al. (2011) may not have performed well on the traditional IAT.

### These results are stable with respect to subsamples

The described results demonstrated stability across subsample analyses. However, faking research has demonstrated that conditions under which faking takes place impact faking behavior (e.g., Röhner et al., 2022). Thus, research has yet to investigate whether the results are also stable with respect to different faking conditions (e.g., IATs measuring different constructs).

### Increasing errors impacts faking detection most strongly

Increasing errors (on the congruent block to fake low scores and on the incongruent block to fake high scores) was revealed to have the strongest impact in the classification process of participants. However, it is important to note that increasing errors was only related to faking success when faking high scores was desired, and not when faking low scores was the goal. Thus, the use of this index helps in detecting successful and unsuccessful faking attempts (at least when faking low scores).

### Combining faking indices somewhat improves faking detection

The combination of faking indices somewhat outperformed the use of single faking indices when contrasted against the overall sample. Nevertheless, combinations did not dramatically enhance classification quality (especially if subsample analyses are also considered). There may be other combinations of indices that could prove optimal and could be investigated in future research.

### Not all behaviors that revealed faking were successful in changing IAT effects as desired

Although the strategy of increasing errors on the congruent block could be used to detect faking low at a level of 84 %, it was not significantly related to faking success. Thus, although fakers of low scores employed this strategy, it did not alter their IAT effects as desired. Note that this result emerged irrespective of how faking success was computed. In other words, increasing errors on the congruent block allows for detection of (unsuccessful) faking attempts, a finding that aligns with Röhner et al. (2013). Furthermore, strategies that indicated fakers differed strongly in their association with faking success (i.e., from small to large correlation effect sizes).

### Not all behaviors that were successful in changing IAT effects as desired revealed faking

Not all strategies that were related to faking success were useful in identifying faking at levels above chance. Although the strategy to reduce errors on the incongruent block was related to a small extent to faking success, when faking low, fakers did not use this strategy above chance levels (i.e., this strategy was successful in changing the IAT effect as desired but was not implemented by most fakers and, thus, was not able to detect faking above chance levels with this strategy). This finding may indicate that this strategy was not possible to be used by fakers. Note that this result emerged irrespective of how faking success was computed. When faking success was computed as an interaction effect, but not when it was computed as *D* change, the strategy to accelerate on the incongruent block was also related to a medium extent to faking success, although when faking low, fakers did not use this strategy above chance levels. The difference with respect to the computation of faking success points to the reduction of noise in assessing faking success, when using interaction effects instead of *D* change, as had been expected. Comparably, when faking success was computed as an interaction effect, but not when it was computed as *D* change, the strategies to accelerate on the congruent block and to reduce errors on the congruent block were related to a small extent to faking success. However, when faking high, fakers did not use this strategy above chance levels.

The Ratio 150–10000 index (Agosta et al., 2011) was always negatively related to faking success. In other words, this behavior was even counterproductive when faking irrespective of how faking success was computed and irrespective of whether high or low scores were faked.

### Properties and limitations of faking indices

Slow_Co, Accel_Co, Slow_In, Accel_In, IncErr_Co, RedErr_Co, IncErr_In, and RedErr_In (Röhner et al., 2013), as well as CTS, require that two IATs are administered to the same test taker. Fakers and non-fakers are then detected by comparing the performance of each test taker in the two IATs. Thus, the application of these faking indices is possible if researchers have data from non-faked and faked IAT performance for each test taker. This may raise concerns about whether these indices could be employed in applied settings. It is very unlikely that fakers in applied settings, when confronted repeatedly with an IAT, will fake the second but not the first IAT. Thus, administering a true baseline assessment would be difficult. Cvencek et al. (2010) previously demonstrated that using an IAT that measures an unrelated construct for which there is no motivation to be faked can be used in such situations to obtain the necessary baseline assessment. They found that, when using a flower–insect attitude baseline IAT, subsequent performance on a child–sex association IAT produced a faking index that identified offenders as pedophiles and non-pedophiles above chance levels. However, the IAT represents a task that relies on the assessment of reaction times and, as such, stimulus-specific effects have been demonstrated to be related differentially with different IATs (e.g. Bluemke & Friese, 2006; Meissner & Rothermund, 2015). Such IAT-specific effects can lead to meaningless increases or decreases in the IAT scores from different IATs that are compared with one another according to the procedure suggested by Cvencek et al. (2010). Thus, the interpretation of absolute *D* changes of different IATs does seem problematic. For applied settings, one would prefer indices that can be computed without a baseline assessment.

In contrast to the other indices, Ratio 150–10000 by Agosta et al. (2011) is based on the administration of a *single IAT* and, as such, does not present the abovementioned limitation. However, in our study and in Röhner et al. (2013), this index was unable to indicate fakers and non-fakers at levels above chance, and it may even assess behavior that is counterproductive to faking. Thus, the application of this index might be restricted to the aIAT.

In practical settings, one rarely has data from two IATs, and as such, a challenge for future research is to focus on faking indices that can be computed on a single IAT. This could be possible with methods such as diffusion modeling. Although not developed to indicate faking, Klauer et al. (2007) suggested that faking affects indices derived from diffusion model analyses. Notably, Röhner and Thoss (2018) and Röhner and Lai (2021) showed that faking was related to changes in participants’ speed–accuracy setting (i.e., IAT_a_) and in non-decision components such as task-switching or motor responses (i.e., $\mathrm{IA}{\mathrm{T}}_{t_0}$; e.g., Schmitz & Voss, 2012). Moreover, recent research has demonstrated that participants’ speed–accuracy setting (i.e., IAT_*a*_) consistently indicates faking on several IATs with the help of machine learning (e.g., Röhner et al., 2022).

### Limitations

Our study has potential limitations regarding the applicability of faking indices. First, we examined only one personality dimension (i.e., extraversion). Future research should investigate the applicability of faking indices using other constructs. Second, our samples consisted primarily of students who had been instructed to fake (or not). Whether results are generalizable to samples from other populations (e.g., forensic samples) and to naturally occurring faking are avenues for future research. Third, participants for our reanalyzed data sets had an average age of 22.05 years. Age is related to reaction times and errors (e.g., Endrass et al., 2012). Thus, future research should look to replicate and extend our findings using samples that are more diverse. Fourth, faking behavior depends on faking conditions (e.g., Röhner et al., 2022). Thus, future research should investigate faking indices in a variety of faking conditions. This would also allow for exploration of when and why indices fail. Lastly, given that researchers do not know which faking strategies are applied by fakers across settings, developing decision trees in order to classify participants as fakers with specific faking strategies (or combinations of them) is a relevant avenue for additional research.

### Conclusion

Applying recommended faking indices in a large sample revealed that faking detection in IATs is a complex endeavor. Fakers of high scores and fakers of low scores use different faking strategies, which aligns with recent theorizing about different faking processes (Bensch et al., 2019; Röhner & Holden, 2021). These results demonstrated stability across subsample analyses. Not all faking indices that have been suggested were able to detect fakers at levels above chance. Not all faking indices were equally important in detecting (successful) faking. Of note, combinations of faking indices may somewhat improve classification accuracy. Further, not all faking behaviors that were employed by fakers were successful in changing their IAT effects in the desired direction. Finally, not all successful strategies were actually used by (most of the) fakers. For these reasons, it is recommended that investigators combine indices depending on the context, and look for their convergence.

## Data Availability

The data and materials for all experiments are available at the OSF (https://osf.io/6vt7c/).

## Footnotes

1 One should note that the individual definition of congruent and incongruent blocks does not need to be the same for all respondents when using the relative difficulty of the blocks for the respondents as the definition criterion for congruent and incongruent blocks. For participants with high scores on extraversion, the congruent blocks and incongruent blocks would be denoted as above, whereas for participants with high scores on introversion, it would be the reverse. To facilitate communication, we use the term congruent blocks if the pairing of attribute discrimination and target discrimination is congruent with high values in the construct that is measured (here, extraversion), whereas we use the term incongruent blocks if the pairing of attribute discrimination and target discrimination is incongruent with high values in the construct that is measured.

2 The categories mentioned were used as attribute categories and target categories in the present extraversion IAT.

3 When using the terms faking of higher scores and faking of lower scores, we are referring to the increases and decreases in the size of the IAT effect (Röhner et al., 2013). In addition, there could be cases in which fakers of the IAT might want to change the direction of their IAT effect (e.g., to show that they prefer Blacks relative to Whites when they actually prefer Whites relative to Blacks; see e.g. Röhner & Lai, 2021).

4 At a baseline assessment, participants completed the IAT under standard instructions.

5 Because faking indices mirror the strategies fakers use in order to fake, the terms faking strategy and faking index can in most cases be used interchangeably. In order to increase understandability and to avoid using both terms in each case, we will use hereafter the term which best fits the meaning within the respective context.

6 Note that Röhner et al. (2013) used the term "unsystematic changes”; however, based on a reviewer’s recommendation, we instead use "effects of repeated measurement.”

7 We used five subsamples based on consideration of the related power and sensitivity (see Method).

8 For the analyses of the five subsamples (i.e., *n* is at least 49 participants each in the faking group and in the control group), the R package pROC version 1.17.0.1 revealed a power > .95 at a significance level of 0.05 for ROC curve analyses to detect an AUC associated with acceptable levels of discrimination (i.e., *AUC* = .70 to .80; see Hosmer & Lemeshow, 2000)

9 For the analyses of the five subsamples (i.e., *n* is at least 49 participants each in the faking group and in the control group), sensitivity analyses using the R package pROC version 1.17.0.1 revealed a minimum detectable AUC of .70.

10 The power was computed after exclusion of outliers with extreme Mahalanobis distances. Power ranged between .998 for the smallest group of participants (*n* = 236) and .999 for the largest group of participants (*n* = 258).

11 Sensitivity was computed after exclusion of outliers with extreme Mahalanobis distances. Sensitivity ranged between *q* = .23 for the smallest group of participants (*n* = 236) and *q =* .22 for the largest group of participants (*n* = 258).

12 The power was again computed after exclusion of outliers with extreme Mahalanobis distances. Power ranged between .95 for the smallest groups of participants (*n* = 236 and *n* = 243) and .96 for the largest groups of participants (*n* = 258 and *n* = 244).

13 Sensitivity was computed after exclusion of outliers with extreme Mahalanobis distances. Sensitivity ranged between *q* = .302 for the smallest group of participants (*n* = 236 and *n* = 243) and *q =* .296 for the largest group of participants (*n* = 258 and *n* = 244).

14 Before undertaking the correlation analyses, we used Mahalanobis distances to identify and exclude outliers. We excluded 54 participants from 5970 cases (i.e., 0.01% of all cases), whereby the term “cases” refers to participants × faking indices.

15 We subtracted the score of the repeated measurement from the baseline when comparing the control group versus the faking low group and subtracted the baseline from the repeated measurement when comparing the control group to the faking high group.

16 The *df*s of all ANOVAs were adjusted according to Greenhouse–Geisser (Weiner et al., 2012).

17 To give more insight into differences between correctly classified participants and failed-to-be-classified participants, descriptive statistics on the respective faking strategies and faking indices are summarized in the Supplement at OSF (https://osf.io/6vt7c/).

18 To give more insight into differences between correctly classified participants and failed-to-be-classified participants, descriptive statistics on the respective faking strategies and faking indices are summarized in the Supplement at OSF (https://osf.io/6vt7c/).
